# Visual mismatch negativity to masked stimuli presented at very brief presentation rates

**DOI:** 10.1007/s00221-016-4807-1

**Published:** 2016-11-03

**Authors:** Maria Flynn, Alki Liasis, Mark Gardner, Tony Towell

**Affiliations:** 1 Department of Psychology, University of Westminster, 115 New Cavendish Street, London, W1W 6UW UK; 2 Department of Ophthalmology, Great Ormond Street Hospital for Children, London, WC1N 3JH UK

**Keywords:** Visual mismatch negativity, Subliminal, EEG, ERPs, Conscious perception

## Abstract

Mismatch negativity (MMN) has been characterised as a ‘pre-attentive’ component of an event-related potential (ERP) that is related to discrimination and error prediction processes. The aim of the current experiment was to establish whether visual MMN could be recorded to briefly presented, backward and forward masked visual stimuli, given both below and above levels of subjective experience. Evidence of visual MMN elicitation in the absence of the ability to consciously report stimuli would provide strong evidence for the automaticity of the visual MMN mechanism. Using an oddball paradigm, two stimuli that differed in orientation from each other, a + and an ×, were presented on a computer screen. Electroencephalogram (EEG) was recorded from nine participants (six females), mean age 21.4 years. Results showed that for stimuli that were effectively masked at 7 ms presentation, there was little variation in the ERPs evoked to standard and deviant stimuli or in the subtraction waveform employed to delineate the visual MMN. At 14 ms stimulus presentation, when participants were able to report stimulus presence, an enhanced negativity at around 175 and 305 ms was observed to the deviant and was evident in the subtraction waveform. However, some of the difference observed in the ERPs can be attributed to stimulus characteristics, as the use of a ‘lonely’ deviant protocol revealed attenuated visual MMN components at 14 ms stimulus presentation. Overall, results suggest that some degree of conscious attention is required before visual MMN components emerge, suggesting visual MMN is not an entirely pre-attentive process.

## Introduction

The automatic detection of change in the visual environment is key to human survival and adaptive behaviour. The mechanisms for detecting changes in the absence of focal attention are beginning to be understood. However, how the brain detects change and orients to stimuli that may then come under focal attention is less well understood.

Current literature suggests that the mismatch negativity (MMN) is an electrophysiological correlate of the brain’s ability to predict changes in environmental regularities. Current interpretations of the neural mechanisms that underlie the generation of the MMN posit the MMN within a predictive coding framework based on Bayesian principles. It is assumed that rather than passively registering environmental regularities the brain actively predicts the causes of sensory inputs (Friston 2005, 2010; Rao and Ballard 1999). Within this framework, sensory input entering the primary visual cortex is actively compared with top–down predictions and the MMN is elicited when there is a failure to suppress error prediction (Friston 2005; Garrido et al. 2009). For a review of MMN studies interpreted within this framework see Stefanics et al. (2014); Winkler and Czigler (2012).

The visual MMN is observed as a negative ERP deflection that usually peaks around 150–400 ms post-stimulus change and is maximal over posterior electrode locations. Recent interpretations of the component structure of the visual MMN suggest an initial negative component occurring between 150 and 200 ms and a later negative component between 200 and 400 ms (Kimura 2012). Generators have been localised to visual extrastriate areas and prefrontal areas (Kimura et al. 2010; Urakawa et al. 2010). The MMN is best recorded in the absence of focused attention, because during focal attention the electrophysiological signature of the visual MMN is overlapped by other negative ERP components in the same latency range.

The relationship between the elicitation of visual MMN and allocation of attentional resources is a controversial area in MMN research. The issue of how the automaticity of the MMN generation can be characterised is still to be understood (Kimura 2012; Rissling et al. 2013). Although it has been proposed that generation of the visual MMN is an automatic process operating outside the focus of active attention, this has proved difficult to establish empirically. Within the visual system, it is difficult to design a methodologically adequate ‘ignore’ condition due to vision’s primacy in directing continuous behaviour (Czigler 2007; Kimura 2012). Therefore, an issue when interpreting visual MMN research is the difficulty assessing the extent to which attentional resources have been directed to the stimuli irrelevant to the behavioural task. It is unclear in the visual MMN whether it is elicited outside the focus of active attention or whether the N1-/N2-like waves observed in the subtraction waveforms demonstrate the same degree of automaticity as the auditory MMN. In the auditory domain, the MMN can be recorded during sleep (Nielsenbohlman et al. 1991) and in patients in a coma (Kane et al. 1996), suggesting it is an automatic detection of stimulus change, that does not require conscious attention. However, there has been some debate in recent years as to whether even the auditory MMN is truly elicited outside the focus of active attention (Haroush et al. 2010; Rissling et al. 2013), as attentional manipulations have been shown to lead to increases (Restuccia et al. 2005) and decreases (Yucel et al. 2005) in MMN amplitude. Paavilainen (2013) suggested that the elicitation of the visual MMN in the absence of the ability to consciously report the changes in the deviant would provide strong support for the automaticity of the visual MMN mechanism and would be suggestive of pre-attentive cognitive operations in vision.

In many studies, evidence of the pre-attentive nature of visual MMN is sought by the manipulation of the attentional resources of the participant away from task irrelevant stimulus sequences that are of interest to the experimenter, by asking the participant to focus their attention on a second task. For example, irrelevant stimuli are presented peripherally whilst the participant is asked to focus their attention in the middle of the visual field or to focus on an auditory task whilst ignoring visual stimuli presented (Astikainen et al. 2008; Astikainen and Michie 2004; Clery et al. 2013; Czigler et al. 2002; Czigler and Pato 2009; Kenemans et al. 2003; Kremlacek et al. 2006; Stagg et al. 2004; Stefanics et al. 2011; Tales et al. 1999, 2002); using visual illusions to divert attentional resources to the illusory stimulus (Flynn et al. 2009); presenting stimuli that are irrelevant to the task in the peripheral visual field whilst participants focus their attention on the centre of the visual field (Sulykos and Czigler 2011). For a review of tasks used to manipulate attention to events evoking the visual MMN see Stefanics et al. (2014).

There is general agreement that stimuli that are not accessible to conscious awareness (subliminal) can still be analysed. The question of whether a visual MMN can be elicited by deviant stimuli that are reported as ‘unseen’ may provide insight into the relationship of the visual MMN on attention. Studies such as Hsieh and Colas (2012) have shown that stimuli that are not consciously detected can still be analysed and influence perceptual and cognitive function, for a review of similar evidence, see Lin and He (2009). Much of the current debate has moved from whether subliminal stimuli can be perceived, to identifying the nature of the processing that can be achieved without awareness. In particular, within non-conscious processing some have argued for a transient ‘preconscious’ state where information is potentially accessible, yet not accessed. For conscious perception, both bottom–up stimulus strength and top–down attentional amplification are jointly needed but they might not always be sufficient for a stimulus to cross the threshold for conscious perception that can only be evaluated by subjective report (Dehaene et al. 2006).

A number of studies have used subliminal methods to explore ERP components to stimuli that have no emotional content. For instance, in a group of thirteen patients with intractable epilepsy, ERPs were recorded directly from the cortex to stimuli that were presented below and above subjective threshold of awareness (Brázdil et al. 2001). A yellow X (target) and a yellow O (standard) were presented on a white background within a visual oddball paradigm, and the subjective threshold was predetermined for each participant by altering the level of contrast of the stimuli until the participant was no longer able to distinguish stimuli from one another. In the first phase, the stimuli were presented for 200 ms duration (supraliminal condition) and subjects had to button press on detection of the target and a P3 response was recorded. In the second phase of the experiment, the supraliminal stimuli were interspersed with stimuli presented for 10 ms duration (subliminal condition). Analysis of the ERPs evoked to the subliminal target stimuli revealed a P3 component corresponding to that recorded to the supraliminal stimuli, although it was smaller in amplitude and earlier in latency (peaking at 258 ms in the subliminal condition and 391 ms in the supraliminal condition). Brázdil et al. (2001) interpreted these results as implying that perception of the stimuli and higher level processing could occur even if the participant was unaware of the information, but concluded that the P3 evoked in the subliminal condition reflects conscious discrimination even if the participant was unaware of it.

In another study, Bernat et al. (2001a) presented the words LEFT and RIGHT in a counterbalanced oddball design, subliminally for 1 ms. The findings confirmed that a P3 component was significantly greater for the less frequent (either LEFT or RIGHT) than for the frequent stimulus across electrode locations Fz, Cz and Pz, suggesting that the oddball P3 could be recorded to subliminal stimuli. These studies demonstrate that ERPs to stimuli presented below levels of subjective detection can be elicited in the absence of focused attention.

Masking of visual stimuli to investigate the automaticity of brain processes follows the premise that whereas automatic processes can be triggered by both conscious and unconscious stimuli, processing only occurs automatically for unconscious perception. The problem here is assessing the extent of masking and influence of top–down processing which can in turn trigger cognitive processes that can influence decision and action (Kiefer et al. 2012). Czigler et al. (2007) conducted a series of experiments using a visual oddball paradigm to present green/black and red/black checkerboard stimuli that were followed by a mask with stimulus onset asynchronies (SOAs) varying between 14 and 174 ms. A behavioural task varied in the experiments between detecting changes in the size of elements of a central fixation cross and detecting the deviant stimulus. At test (standard or deviant) to mask SOAs longer than 27 ms, deviant stimuli elicited an occipital negative component within the latency range 124–132 ms (visual MMN) with no amplitude increase beyond 40 ms SOA. The detection of deviant checkerboard patterns improved up to 174 ms SOA implying the processes underlying visual MMN elicitation cannot fully explain the overt detection of visual deviance.

Kogai et al. (2011) in a magnetoencephalography (MEG) study presented vertical grating stimuli that varied between standard, deviant and mask in terms of spatial frequency, with differences in spatial frequency such that it was difficult to distinguish the standard from the deviant. An oddball sequence, in which masked stimuli were presented for 433 ms interspersed with standard and deviant stimuli presented for 17 ms, was carried out. During stimulus presentation, the participants task was to respond by pressing a button on detection of the deviant. Behavioural results suggested that the participants could not consciously detect the difference between standard and deviant stimuli. A response to deviant stimuli that was significantly larger than to standard stimuli was recorded in the latency period 143–153 ms, suggesting an automatic response analogous to an MMN could be recorded to masked visual stimuli for changes to spatial frequency, despite participants inability to report this change.

Meng et al. (2015) in an ERP study investigating subjective visual acuity used a three-stimulus visual oddball paradigm to present participants with optotype stimuli, that changed in orientation, in the centre of the visual field at three threshold levels defined by each participants subjective visual acuity: supra-threshold, threshold and sub-threshold. Participants were presented with the stimuli and at the same time were given an active listening task to divert their attention from the visual stimuli. Visual MMN components were found for threshold and supra-threshold conditions, but no visual MMN was elicited when stimuli were presented sub-threshold.

Lastly, in a study utilising the attentional blink paradigm, visual MMN to a deviant visual stimulus emerged in periods of attentional blink and did not differ in morphology or amplitude with visual MMN recorded outside of the attentional blink window demonstrating that the visual MMN is elicited without attentional allocation (Berti 2011).

Overall, these studies which have produced mixed results have attempted to manipulate access to visual information by a combination of masking, stimulus strength, duration and location to test the relationship of visual MMN and attentional allocation. We rationalised that according to the taxonomy of Dehaene et al. (2006), a stimulus of very short duration (at the limits of our technical parameters), presented in a paradigm with no obvious task demands and no subjective report of perception, would satisfy the criteria of subliminal testing. The current experiment extended the research of Flynn et al. (2009) by using a backward and forward masking paradigm to test whether visual MMN could be elicited to stimuli that differed in orientation presented below the subjective threshold of perception. Flynn et al. (2009) in a three-stimulus oddball paradigm, in which one of the infrequent stimuli formed a Kanizsa square that was used to capture spatial attention in place of a behavioural task, revealed visual discrimination responses at occipital electrodes, representative of visual MMN components. Therefore, a novel aspect to the current experiment was that no behavioural task was required of the participant. Evidence of visual MMN elicitation in the absence of the ability to consciously report stimuli would provide strong evidence for the automaticity of the visual MMN mechanism.

## Methods

### Participants

With ethical approval and informed consent, 9 healthy student adults were recruited for the experiment with a mean age 21.4 years, range 18–30 (6 females). Participants reported no history of neurological disease and had normal or corrected-to-normal visual acuity.

### Stimuli and procedure

**Fig. 1:**
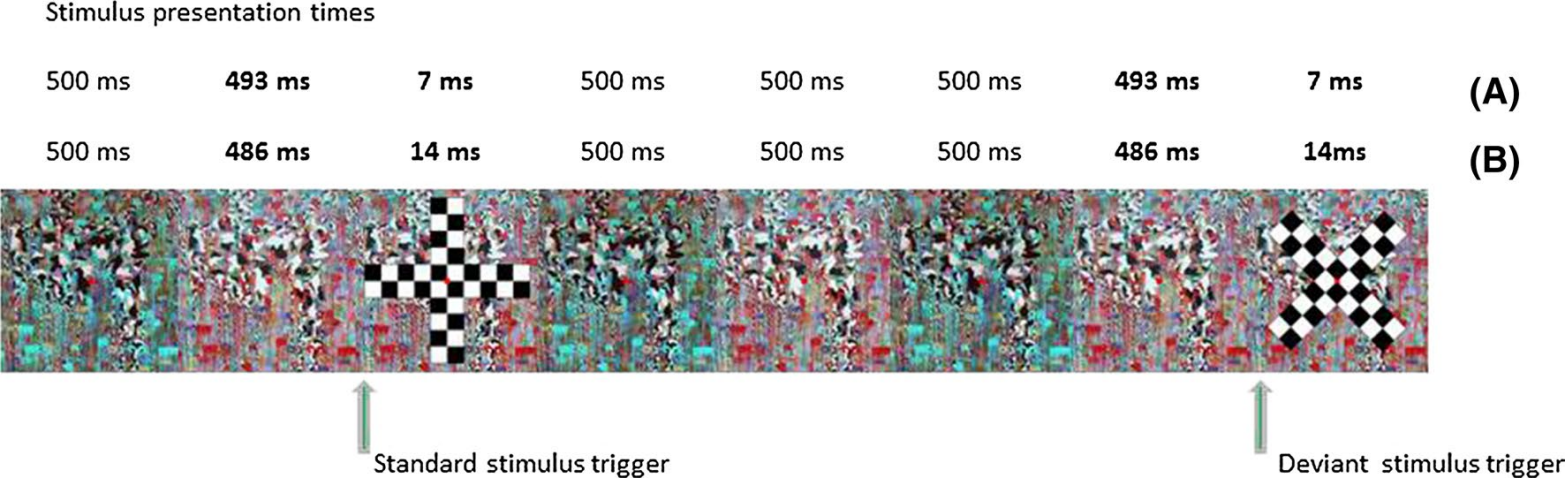
Schematic representation of single stimulation cycle presented in the oddball paradigm. *A* and *B* show stimulus presentation times for 7 and 14 ms, respectively

Two stimuli, comprising of black and white checkerboard elements, differing from each other only in terms of their orientation to form either a + or an ×, were presented in a behaviourally silent oddball paradigm where the ratio of standards to deviants was 8:2. The stimuli were embedded between masking stimuli consisting of complex images whose colours inverted. The background of the standard and deviant stimuli consisted of the same complex image as the mask. The two stimuli were presented at very brief presentation times, below levels of subjective awareness for 7 ms in the first instance and then above levels of subjective awareness at 14 ms. The masking stimuli were presented for between 486 and 500 ms, and details of stimuli, stimulus sequence and durations during experiment are illustrated in Fig. 1 for conditions A and B. The test to mask SOA was determined by the computer refresh rate and was 7 ms.

All stimuli were presented on a 21-inch cathode ray tube (CRT) monitor (Samsung Sync Master), with an NVIDIA GeForce 8800GT 320 MB graphics card, running with a screen refresh rate of 160 Hz. The stimulus presentation software (E-Prime V2.0 Psychology Software Tools Inc.) provided markers to be used during averaging of the electroencephalograph (EEG) to produce evoked potential waveforms.

Participants were seated comfortably in a darkened room 1 m away from the computer screen and requested to fixate on a small red dot in the centre of the screen that was present throughout recording. Within the oddball paradigm, stimuli were presented in a pseudo-random sequence ensuring deviant stimuli were interspersed with standard stimuli. Five blocks of the 7 ms duration stimuli were presented. Each block contained 500 stimuli (400 standards 100 deviants). This was followed by one block of 100 stimuli where the masked deviant (the ×) was presented alone, i.e. with no standard stimulus. The same procedure was then carried out for the 14 ms duration stimuli. There was a break of 1 min between blocks. Following the first block of the 14 ms presentation, participants were asked if they observed anything different from the earlier presentations and prompted to describe what it was. All nine participants reported seeing the stimuli at 14 ms and were therefore included in the current study.

### EEG data recording

Silver–silver chloride electrodes were used to record the EEG activity and were positioned at sites in accordance with the International 10–20 system (Jasper 1958) (Fz, Cz, Pz, Oz, O1, O2, VEOG, M1, M2). The reference and the ground electrode were placed at the right and left mastoid, respectively. An electrode was placed above the left eye to enable online artefact rejection of eye blinks. Continuous EEG was collected using Neuroscan SCAN version 4.3; Compumedics USA, Ltd., El Paso, TX, USA at a sampling rate of 1000 Hz, with a low pass of 100 Hz and a high pass of .05 Hz and stored on a computer for offline analysis.

### VEP data analysis

Continuous EEG data were epoched offline −100 ms pre-stimulus to +500 ms post-stimulus. The epochs were digitally filtered with a band pass 1–30 Hz and baseline corrected employing an average of a 100 ms pre-stimulus baseline as zero. Epochs containing transients greater than ±100 μV were excluded from further analysis. For each participant, ERPs were averaged separately for standard and deviant stimuli, the data re-referenced to Fz, and grand average waveforms were constructed.

From the grand average waveforms, MMN-like differences were identified on the basis of known negative polarity, known emergence over posterior electrode positions and typical latency range 150–400 ms (Kimura 2012). In both conditions, the maximal difference between ERPs to standards and deviants was identified at 175 ms and 305 ms post-stimulus presentation at occipital sites and a 40 ms time window was centred at these latencies for electrodes O1 and O2 (Czigler et al. 2007).

In addition, to delineate the visual MMN, subtraction waveforms were constructed of deviant minus standard and deviant minus deviant alone. The ‘alone’ or ‘lonely’ deviant condition acts as a control for stimulus differences and involves presentation of the deviant stimulus as the only stimulus in a repetitive sequence. The evoked response to the deviant stimulus in context (i.e. within the oddball paradigm whereby a series of standard stimuli are presented) was compared to the evoked response to the same stimulus when presented alone. If a MMN is present, a relative negativity will be apparent only in the evoked response elicited in the context of the oddball paradigm and will not be present when the deviant is presented alone (Kraus et al. 1995).

Mean amplitudes for the time windows were calculated relative to the mean voltage of a 100 ms pre-stimulus baseline for each participant for the standard, deviant and deviant alone stimuli. The mean amplitudes were analysed using *t* tests as outlined below.

## Results

### VEP data analysis

**Fig. 2:**
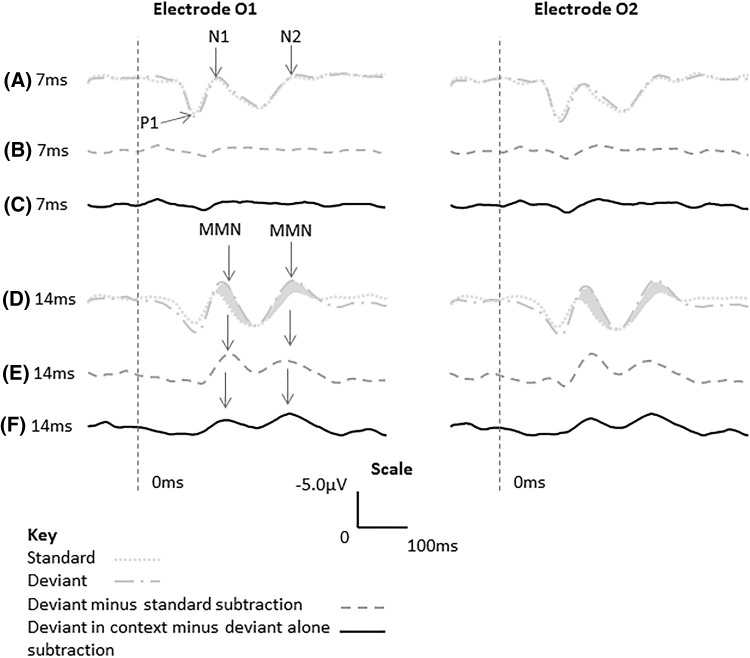
Grand average waveforms referenced to Fz (negative upwards) at electrodes O1 and O2. (*A*) and (*D*), standard and deviant waveforms at 7 and 14 ms duration, respectively, (*B*) and (*E*) deviant minus standard subtraction waveforms at 7 and 14 ms duration, respectively, (*C*) and (*F*) deviant in context minus deviant alone subtraction waveform at 7 and 14 ms duration, respectively. Note the discrimination responses highlighted in *grey*

A visual response was recorded in all participants consisting of a P1-N1-P2-N2 waveform for the stimuli presented at 7 and 14 ms duration. All participants were unable to report the appearance of the + and × stimulus at 7 ms stimulus duration but were able to report the appearance of the + and × at 14 ms. Grand average waveforms were constructed for the standard and deviant stimuli (see Fig. 2a, d, 7 and 14 ms, respectively) for waveforms at electrodes O1 and O2. Visual inspection of the grand average waveforms reveals an enhanced negativity in the ERP response to the deviant when stimuli were presented for 14 ms, with a maximal difference at approximately 175 ms and at around 305 ms compared to the standard stimuli. This amplitude difference was not apparent in the 7 ms condition. A 40 ms time window was centred at each of these latencies for electrodes O1 and O2. For all participants, mean amplitudes for these time windows were calculated relative to the mean voltage of a 100 ms pre-stimulus baseline for standards, deviants and deviant ‘alone’ stimuli.

Visual inspection of the difference waveform of deviant minus standard revealed discrimination components only when the stimuli were presented for 14 ms (Fig. 2e) peaking at approximately 175 and 305 ms, with an amplitude in the order of 2.2 and 1.5 µV, respectively, these were not apparent in the 7 ms condition (2b). In addition, visual inspection of the difference waveform of deviant in context minus deviant presented alone (Fig. 2f) revealed reduced discrimination components, suggesting that some, but not all, of the difference observed was due to stimulus characteristics.

### Statistical analysis of amplitude data

As no differences were apparent between O1 and O2 electrodes following visual inspection, the mean amplitude data for these bilateral occipital electrodes were combined. Data were analysed using paired *t* tests, deviant with standard and deviant with deviant alone for each stimulus duration (7 and 14 ms) and for each time window (early 155–195, late 285–325). Bonferroni corrections were applied. Results showed that there was an increased negative amplitude to the deviant stimulus compared to the standard stimulus at 14 ms stimulus duration for the early time window (*t* = 6.107, d*f* = 8, *p* < .001) and for the late time window (*t* = 4.109, d*f* = 8, *p* = .003) that was not apparent in other conditions (see Table 1).

**Table 1:** Summary of the mean stimulus amplitudes (µV), standard deviation (SD) and paired *t* test comparisons of the grand average waves for standard, deviant and deviant alone stimuli in the early (155–195 ms) and late (285–325 ms) time windows for 7 and 14 ms stimulus duration (ms)

Grand average wave mean amplitudes (µV) and SD for the *early and late* time windows	Stimulus duration (ms)	*t*	*p* (2-tailed*)*
*Time window* 155–195 ms			
Standard (2.11 ± 2.24) and deviant (1.46 ± 1.61)	7	1.569	.155
Standard (1.39 ± 2.74) and deviant (−1.46 ± 2.64)	14	6.107	<.001**
Deviant (1.46 ± 1.61) and deviant alone (1.52 ± 2.77)	7	.082	.936
Deviant (−1.46 ± 2.64) and deviant alone (−.51 ± 1.78)	14	1.279	.237
*Time window* 285–325 ms			
Standard (.58 ± .97) and deviant (.078 ± .93)	7	1.589	.151
Standard (−.31 ± 2.17) and deviant (−2.53 ± 2.92)	14	4.109	.003*
Deviant (.078 ± .93) and deviant alone (−.09 ± 1.43)	7	.427	.680
Deviant (−2.53 ± 2.92) deviant alone (−.72 ± 2.41)	14	2.210	.058

All d*f*’s = 8

* *p* < .01, ** *p* < .001

## Discussion

The results of this study show that for stimuli that were not reportable using the backward and forward masking paradigm employed, i.e. those presented at 7 ms, there was little variation in the ERPs evoked to standard and deviant stimuli. When stimuli were presented at 14 ms duration, participants were able to report the appearance of the standard and the deviant stimuli and the deviant stimulus produced an enhanced negative amplitude compared to the standard stimulus at approximately 175 and 305 ms, respectively. Comparison of the deviant with the deviant alone waveforms using paired *t* tests did not reach significance. However, the deviant minus the ‘deviant alone’ subtraction waveform showed an attenuated response, suggesting that some, though not all, of the differences observed between the standard and deviant responses was due to stimulus difference. The ERP waveforms and the statistical analysis suggest that discrimination responses, possibly reflecting visual MMN components, were recorded in the 14 ms stimulus condition. Overall therefore, these results suggest that visual MMN was not elicited when participants could not report seeing the stimuli but that visual MMN components began to emerge when participants were able to report seeing the stimuli.

The current study suggests that conscious perception of the stimuli was required before visual MMN components could emerge. These results contrast with those of Kogai et al. (2011) who used an oddball sequence in which masked grating stimuli were presented for 433 ms, interspersed with standard and deviant grating stimuli presented for 17 ms. They reported that despite behavioural results suggesting that the participants could not consciously detect the difference between standard and deviant stimuli, a MEG response to deviant stimuli that was significantly larger than to standard stimuli emerged. They interpreted this as suggesting an automatic response analogous to an MMN could be recorded to masked visual stimuli for changes to spatial frequency. Kogai et al. (2011) results raise the possibility that MEG offers a more sensitive methodology with which to investigate visual MMN in the absence of awareness.

A number of studies have reported recording other ERP components to subliminal stimuli. Bernat et al. (2001a) reported a significant parietal P3 to stimuli presented below objective detection threshold levels. Brázdil et al. (2001) reported an ERP to subliminal stimuli that corresponded to the P3 evoked to the supraliminal stimuli. It was, however, smaller in amplitude and earlier in latency in the subliminal condition. A study by Bernat et al. (2001b) showed that a subliminal P3 ERP could be elicited to emotionally valent words that had a component structure similar to a supraliminal P3, although smaller in amplitude by at least a factor of four. The visual MMN response is smaller in amplitude, often in the region of 1–3 µVs, than that of a P3. One explanation for a lack of visual MMN in the 7 ms condition could be that scalp-recorded EEG methods may be relatively insensitive to an attenuated response in the subliminal condition, compared with intracranial EEG or MEG.

Recent interpretations of the component structure of the visual MMN suggest an initial negative component occurring between 150 and 200 ms and a later negative component between 200 and 400 ms (see Kimura 2012). These components, corresponding to visual N1 and N2, respectively, are apparent in the present experiment. The early visual MMN component, in the N1 latency period, is thought to be due to differential activation of afferent neuronal populations between stimuli, thus reflecting their state of habituation. Differences in habituation are thought to be due to differences in stimulus probability and, as such, the amplitude of VEPs evoked to deviant stimuli is larger than those evoked to standard stimuli (Kimura 2012). Several studies have suggested that the enhanced negativity observed in the deviant minus standard subtraction waveforms in the N1 latency period is therefore due to the refractory state of the neurons due to the rareness of the deviant stimuli in comparison with the standard stimulus (Kenemans et al. 2003; Mazza et al. 2005). By contrast, other studies have interpreted this difference in the N1 latency period as a genuine visual MMN response (Czigler and Sulykos 2010).

The late visual MMN component, in the N2 latency period, is thought to be representative of sensory memory formation or prediction error responses that are generated when a current event is incongruent with events predicted on the basis of sequential regularities (Kimura 2012; Kimura et al. 2011). Studies incorporating an ‘equiprobable paradigm’ specifically designed to separate the effects of refractoriness and sensory memory/prediction error responses elicit enhanced negativities in the latency periods observed in the current 14 ms condition (Czigler et al. 2006; Kimura et al. 2009).

In the current experiment, the use of the same stimulus only changing its orientation was used to control for habituation. However, some of the changes observed may be due to the activation of fresh neuronal populations within the oddball condition. When the ERP response to the deviant stimulus presented alone and out of context was subtracted from the deviant ERP response to the deviant presented in the oddball paradigm, no significant differences were revealed when analysed by paired *t* test. This, in combination with the observed reduction in the subtraction waveform (Fig. 2f), suggests that some of the difference observed in the 14 ms condition may be due to stimulus characteristics. Although the stimuli used as standard and deviant were the same, only changing in orientation, stimulus differences cannot be ruled out. These, however, may be reflected in the enhanced negativity in the N1 latency period.

In summary, the current experiment used a backward and forward masking paradigm to investigate discrimination processes in stimuli that changed in orientation by employing an oddball paradigm to establish whether a deviant stimulus could elicit registration of stimulus discrimination that was independent of the ability to report that stimulus. The emergence of visual MMN in the absence of the participants ability to report changes in the deviant stimuli would provide strong evidence for pre-attentive processes in visual cognition. However, when participants could not report seeing the masked stimuli (at 7 ms presentation) no visual MMN was recorded. Evidence of visual MMN components only appeared when the masked stimuli were presented for 14 ms and the participants were able to report their appearance, although some of the difference between the standard and deviant ERPs was accounted for by stimulus difference. The present study therefore revealed no evidence for the automaticity of the visual MMN.

As proposed by Dehaene et al. (2006), a continuum of subliminal states may exist, depending on masking strength, top–down attention, and task instructions. If stimulus strength is particularly strong, in the absence of top–down attention, preconscious processing is said to occur. Therefore, given that MMN is sensitive to linguistic variables (Casado and Brunellière 2016; Scharinger et al. 2016) and emotional expression (Liu et al. 2016; Schirmer et al. 2005), it may be too simplistic to describe it in terms of a pre-attentive automatic response but rather a response to a sophisticated dynamic memory system operating at stages before and around the threshold of conscious awareness.
